# A Consideration of Alternative Sample Spaces Used in Coin-Toss Problems

**DOI:** 10.1007/s42330-022-00224-3

**Published:** 2022-09-01

**Authors:** Amy Renelle, Stephanie Budgett, Rhys Jones

**Affiliations:** 1 Department of Statistics, University of Auckland, Auckland, New Zealand; 2 Faculty of Health and Medical Sciences, University of Surrey, Surrey, UK

**Keywords:** Statistics education, Sample spaces, Heuristics, Randomness

## Abstract

This paper examines coin-toss comparison questions from two recent studies involving undergraduate students and high school teachers and connects to findings from two prior studies in the literature. Considering possible sample spaces employed by participants, this is a reflection on whether one sequence could be more likely depending on the interpretation of the question. To critique the choice of sequences and determine possible scenarios in which one sequence may be more likely than the other, three alternative sample spaces were explored. It was determined that different sample spaces can lead to one sequence being more likely to occur than the other. Further evaluation discusses whether alternative sample spaces may have been utilised by the participants in each of the studies, and hence, the paper concludes with an advocacy to enquire deeper into participants’ reasoning when investigating coin-toss questions.

## Résumé

Cet article s’intéresse aux aspects liés à la comparaison de tirages au sort dans deux études récentes menées auprès d’étudiants de premier cycle et d’enseignants au secondaire et nous établissons des liens avec les conclusions de deux études antérieures consultées dans la documentation. En tenant compte des espaces échantillon potentiels utilisés par les participants, cet article amène une réflexion à savoir si une séquence peut s’avérer plus probable selon l’interprétation faite de la question. Afin d’évaluer le bien-fondé des séquences choisies et pour déterminer les scénarios possibles dans lesquels une séquence serait plus susceptible d’être présente qu’une autre, nous avons exploré trois espaces échantillon différents. On a jugé que des espaces échantillon distincts peuvent donner matière à ce qu’une séquence est davantage susceptible de se matérialiser qu’une autre. La suite de l’évaluation porte sur la question de savoir si des espaces échantillon différents peuvent avoir été utilisés par les participants dans chacune des études et de ce fait, la conclusion de l’article revendique un examen plus approfondi du raisonnement des participants lorsque l’on étudie des questions liées au tirage au sort.

## Introduction

Frequently, researchers will test participants’ randomness perceptions by asking them to choose, from a set of sequences, which sequence they believe to be most or least random. Literature (e.g., Kahneman & Tversky, 1972; Tversky & Kahneman, 1974) has commonly found that participants tend to respond incorrectly, holding a non-normative view (overlooking independence of events) or reasoning incorrectly. In this paper, two questions that compare coin-toss sequences are analysed. Three possible sample spaces are explored: the ratio of heads and tails, the length of the longest run, and the number of alternations or switches in a sequence. Deliberation of participant interpretations of coin-toss questions is important for considering the presence of randomness misconceptions. There is a clear need for researchers to reflect upon the likelihood of participants having utilised a certain interpretation through questioning participant justifications. Following the reflection on the pilot study, research into New Zealand secondary school teachers’ potential use of alternative sample spaces that forms part of the first author’s doctoral thesis is examined. Further examples are then considered to exemplify how alternative sample spaces may provide appropriate reasoning for responses. Thus, the purpose of this paper is to reflect on results from a small pilot study, debate whether participants from the pilot study and three other studies may have been utilising alternative sample spaces, and advocate research investigating further the possibility of alternative sample spaces being used.

## Background

Randomness is one concept prone to judgment using the heuristics and biases set out by Kahneman and Tversky (1972) in their landmark paper. Their paper described a set of judgmental heuristics and biases frequently employed when making decisions under uncertainty, and since then, a wealth of research has shown that students, teachers, and everyday citizens can all exhibit these biases. This paper will begin with an examination of data from student participants (undergraduates in the pilot study) and responses from secondary school teachers (mathematics and statistics teachers in the thesis research). This is followed by two further examples with undergraduates and high school students. As noted, teachers may also use heuristics and biases and this could be passed on to their students (see Arteaga et al., 2010; Batanero et al., 2014). It may be the case that attending to teacher perceptions could minimise the occurrence of misconceptions among students and, therefore, within the general population.

Chernoff (2009), however, suggested that the presence of randomness misconceptions could be overstated as it is possible some participants may be using alternative sample spaces when answering coin-toss comparison questions. Focused on sequences derived from flipping a fair coin five times, Chernoff considered possible subjective sample spaces with the aim of understanding participants’ responses. In the first study, participants (56 prospective teachers) were given a coin-toss question asking them to choose which sequence was least likely. Responses indicated that participants primarily focused on the ratio of heads to tails. Chernoff further noted that the sequences may also be ranked in terms of their longest run or the number of switches. The study was repeated with a second set of participants (239 prospective teachers), who were given a different set of sequences that all had the same ratio of heads to tails. Eighty-two percent correctly responded that all sequences were equally likely to occur. Observing that this does not necessarily mean that the participants had correct reasoning underlying their choice, Chernoff reviewed participants’ justifications. Summarising his findings, he indicates that, “…there exists reason to suspect individuals answer the task according to a subjective-sample-space” (p. 107).

To provide an understanding of their responses, reflection on participant justifications is important (Shaughnessy, 1992). Conducting similar research to Kahneman and Tversky (1972, 1974), Shaughnessy (1977) investigated the use of heuristics to answer probability questions and suggested that it was necessary to include the option of equally likely sequences and query participant thinking in order to obtain more accurate data. By asking participants to provide reasoning for their responses “…it was possible to gain some insight into the thinking process of the students as they answered the questions” (p. 308). For example, when considering one student’s reasoning, it could be seen that the normative response was supported with incorrect reasoning that both sequences were equally likely “because each outcome has 3 boys and 3 girls” (p. 310). Hence, this indicated that some participants may have been using the representativeness heuristic (Kahneman & Tversky, 1972, 1974) despite getting the correct answer.

Our research question is then as follows: *which sample space (alternative or normative) seems likely to have been used by participants based on a reflection of their responses?*

## Pilot Study

With the aim of investigating students’ randomness perceptions, a small exploratory study presenting various experiences of randomness was conducted (Renelle et al., 2019). A small selection of students who had recently completed a first-year probability course at the University of Auckland were contacted via email and invited to participate. From the replies to this and availability, two participants were recruited for the focus group, one identifying as male and one as female.

The pilot study consisted of a pre-task, main task, and post-task. The pre-task was an exercise worksheet completed individually by the participants before the main task, without guidance. Likewise, the post-task was also an exercise sheet completed individually, without guidance, after the main task. After completing each of these tasks, the researchers queried participants’ responses. During the main task, the participants interacted with a prototype digital tool called the *Scampy Tool* (Budgett & Pfannkuch, 2018). One mode of the tool with which the participants interacted can be viewed at: https://tinyurl.com/y2zdupzv. Using a “think-aloud” protocol, the participants verbalised their ideas as they progressed through an activity using the tool with the researchers, the first and second authors, probing as to their reasoning for their actions and choices. Although the main task and the *Scampy Tool* were central parts of this research, for this paper, the pre-task and post-task assessments are of most interest.

The pre-task and post-task assessments were used to determine if any changes to participants’ perspectives had occurred after using the tool. After completion of both the pre-task and the post-task, the participants were asked to articulate their reasoning for the choices they made.

Questions 1 and 2 of the pre-task and post-task assessments asked:

Which of the following do you think is more random?

Question 1: HTTHTHTHHT or HHHTTHHTTT

Question 2: HTTHHTHTTH or HTTTTTHHTH

In both questions, the first sequence was contrived by the first author and the second was randomly generated. Responses for both participants are shown in Table 1.

**Table 1:** Pre-task and post-task responses of participants 1 and 2

		Participant 1		Participant 2	
		Sequence 1	Sequence 2	Sequence 1	Sequence 2
Pre-task	Question 1	✔		✔	
	Question 2	✔			✔
Post-task	Question 1		✔	✔	
	Question 2		✔	✔	

When creating the contrived sequences, the main aim was to have short runs and many switches, an approximately equal number of heads and tails, and a slight pattern. For example, question 1, sequence 1 has a repeating pattern in the middle of the sequence: TH-TH-TH. It also begins with HTTH and ends with THHT (inverse patterns). By comparison, the randomly generated sequences have longer runs and alternate less frequently. There may still be a perceivable pattern, however. For example, in question 1, sequence 2 could be sectioned into a pattern like this: HHH-TT-HH-TTT. Sequences of length ten were deemed appropriate for the pilot study where the focus is on the length of runs and number of alternations; random sequences can still be patterned, particularly when they have only ten observations.

Three sample space partitions are explored; the ratio of heads and tails, the longest run, and the number of switches (Chernoff, 2009). Table 2 gives the probabilities of both sequence 1 and sequence 2 occurring under all three sample space partitions, with the more likely sequence identified in bold. See the Appendix for the calculation of these probabilities.

**Table 2:** The likelihood of sequences 1 and 2 for questions 1 and 2 under three alternative sample spaces

	Sample space partition used					
	Ratio of heads and tails		Longest run		Number of switches	
	Sequence 1	Sequence 2	Sequence 1	Sequence 2	Sequence 1	Sequence 2
Question 1	**0.246**	**0.246**	0.172	**0.361**	0.0703	**0.164**
Question 2	**0.246**	0.205	**0.172**	0.123	0.164	**0.246**

Alternative sample spaces can provide a justifiable reason for participants to suggest one sequence is more likely than another to occur. The sample space used by the participants may be determined by examining their explanations.

### Ratio of Heads and Tails

Considering their pre-task assessment responses, participant 1 may have been using this alternative sample space because they selected sequence 1 in both questions 1 and 2 in the pre-task (Table 1). Likewise, in the post-task assessment, participant 2 may have been using this alternative sample space.

### Longest Run

To be able to argue that an alternative sample space partition of the longest run may have been used, a participant would have needed to select sequence 2 in question 1 and sequence 1 in question 2. In both the pre-task assessment and post-task assessment, neither participant selected this combination of sequences. Therefore, it would be difficult to argue that either participant selected the most likely sequence using the sample space partition of the longest run.

### Number of Switches

To be able to argue that the sample space partition of the number of switches may have been used, a participant would have needed to select sequence 2 in both question 1 and question 2. In light of their post-task assessment responses, participant 1 may have been using this alternative sample space.

### Participants’ Verbal Reasoning

Does the participants’ verbal reasoning for their choice in sequences give us any insight as to whether an alternative sample space may have been used? While the participants’ answers to the pre- and post-task questions are not enough to know exactly how they are reasoning, probing their verbal explanations might provide more insight into their choices. The keyword used by both participants when asked about their choice of sequences was *pattern*. This excerpt, from the probing questions that followed participants’ completion of the pre-task assessment, exemplifies the conversation around sequence choices and patterns:

I: … So just an explanation as to why you chose the sequence that you chose?

P2: Umm I guess I would say for this one [question 1; sequence 2], there’s like sort of a pattern, like 3, 2, 2, 3… and for this one… I’m not sure, I’m just, like it just seems more random to me [question 2; sequence 2].

P1: Yup, I [agree]… that when there’s more pattern, it seems less random. I mean, I assumed that either heads or tails, and when it’s completely random, then it should be pretty—heads should be followed by tails, should be followed by heads, or should be rather evenly spaced out… And that seems to be more spaced out [question 1; sequence 1], where that’s a pattern [question 1; sequence 2]. And what she said, there’s accumulation of a whole bunch of H here and a whole bunch of T there. Um, same as [question 2; sequence 2], there’s five Ts in a row that seems to be less random than… when the chance of actual T should be half.

Participant 1 elaborated on their explanation by saying, “…when it’s evenly spaced out, no clusters, that seems to be what random means”. It is difficult to pinpoint what is seen as a pattern to the participants, but from the conversations held, the following is posited as a possible meaning of the term when used by participant 1. As the session progressed, it seemed that this participant’s use of the word *pattern* referred to repetition, although two different kinds of repetition. The first kind of repetition was related to clusters, where only one outcome was repeated, such as TTT (P1: “I used the word pattern by referring to a cluster, clusters of T”). The second kind of repetition was related to alternations, in particular repeating alternations, such as HT-HT (P1: “when it follows a particular order, head, tails, head, tails, head, tails, then that is something more of a pattern to me”). In the post-task assessment, participant 1 amended their previous statement about patterns, “…the patterns [first kind of repetition] and clusters now seem to be more random because when it follows a particular order… then that is something more of a pattern [second kind of repetition] to me”. Participant 2 reasoned similarly.

When interpreting possible meaning behind both students’ reasoning, the use of *pattern* seemed to refer to repeated observations, which could be describing the longest run. By comparison, using *pattern* to refer to alternating sub-sequences could relate to the number of switches in the sequence. This may mean that the participants’ reasoning could represent possible use of alternative sample spaces although, as noted, it is difficult to truly understand the participants’ underlying thinking around patterns. If alternative sample spaces were not used by the participants, it may be that the participants were reasoning heuristically (Tversky & Kahneman, 1974). For example, the participants’ reasoning could have been influenced by the Gambler’s fallacy, whereby the participants’ “…estimate of the probability of tails on a particular toss increases with the number of consecutive heads that preceded that toss” (Tversky & Kahneman, 1974, p. 1130). This may have been what participant 1 was referring to when talking about patterns that alternate often as per their comment from the conversation above, “when it’s completely random, then it should be pretty—heads should be followed by tails, should be followed by heads”.

As such, use of the sample space partition of the longest run seems unlikely as neither participant selected the most likely sequences under this partition. Further, participant 1, in the post-task assessment, may have been using the sample space partition of the number of switches but contradicted this in their verbal reasoning suggesting that the second kind of repetition (relating to alternations) was deemed less random compared to the first kind of repetition (relating to clusters). Additionally, their verbal reasoning did not seem to indicate the use of the sample space partition of the ratio of heads and tails, so this also seems unlikely to have been utilised when selecting the sequences.

With only brief reasoning offered by both participants, it cannot be determined whether a personal sample space had been utilised. Interestingly, neither participant attempted to reason that the two sequences would be equally likely and therefore they did not seem to use a normative approach. An equally likely option was not explicitly offered to participants; using an open-ended question, participants were invited to respond as they deemed most appropriate. This is a potential limitation of the pilot study. While the participants could have suggested that both sequences were equally likely, without this option being presented in the question, it is possible the participants felt this was *not* a plausible response. One development following the pilot study was to include this option explicitly in the thesis study questionnaire.

On reflection, the study highlighted the importance of ascertaining participant thinking as clearly as can be articulated. Intentionally querying participants’ choices and attempting to uncover their thought processes is a necessary step to ensure plausible interpretations of student reasoning can be made (Shaughnessy, 1992). Querying whether alternative reasoning could have been used may uncover a different picture than first expected. However, the participants’ thinking when selecting the sequences in questions 1 and 2 of the pre-task and post-task assessments is not enough to establish a likely path of reasoning. Further, the verbal reasoning offered by the participants in this small pilot study seemed to contradict the use of possible alternative sample spaces. As a counterpoint, however, the participants’ might not have used one particular sample space consistently (Konold et al., 1993), potentially leading to seemingly contradictory reasoning. For example, we could conjecture that the participants may have paid attention to an array of different features of the sequences, hence producing muddled reasoning that appeared inconsistent with some of their sequence selections. It therefore remains unclear whether these participants held randomness misconceptions.

## Thesis Study

Following the pilot study described above, the authors decided to examine the perceptions of randomness held by New Zealand secondary school mathematics and statistics teachers. As noted in the background section, teachers may also hold randomness misconceptions, and these could be passed on to students. However, as previous research has highlighted, it is important to consider whether alternative sample spaces could have been used by participants so as to ensure a more accurate identification of potential misconceptions present within the sample.

**Fig. 1:**
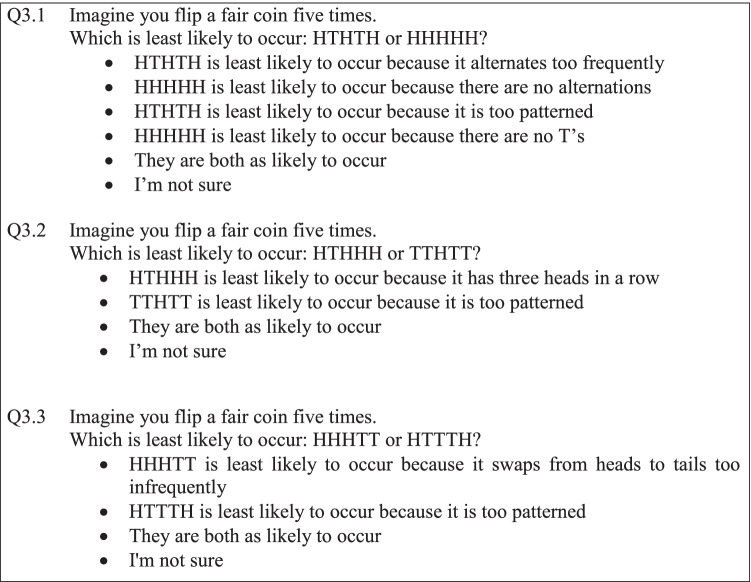
Three coin-toss comparison questions from the questionnaire

New Zealand secondary school mathematics and statistics teachers (*n* = 150) were invited to participate in an online, anonymous questionnaire distributed via an email mailing list. The questionnaire was created in Qualtrics (https://www.qualtrics.com), and participants were recruited through several New Zealand mathematics and statistics associations. To investigate the potential use of alternative sample spaces, three comparison questions were asked (Fig. 1).

This immediately followed demographic information questions, so were the first randomness-based questions the participants encountered. As in Fig. 1, participants were invited to select a descriptive response that aimed to clarify participants’ thinking. Qualitative responses were not practical in this initial questionnaire due to the sample size, so these descriptive responses helped to bridge the gap between understanding participants’ reasoning and obtaining a reasonable number of responses.

As seen in Table 3, the sequences were specifically chosen to assist in the identification of potential alternative sample spaces being used. Sequences of length five were deemed appropriate for the thesis study due to comparability to Chernoff’s (2009) findings.

**Table 3:** The likelihood of sequences 1 and 2 for questions 3.1, 3.2, and 3.3 under three alternative sample spaces, with the more likely sequence identified in bold

	Sample space partition					
	Ratio of heads and tails		Longest run		Number of switches	
	Sequence 1	Sequence 2	Sequence 1	Sequence 2	Sequence 1	Sequence 2
Q3.1: HTHTH and HHHHH	**0.3125**	0.03125	0.0625	0.0625	0.0625	0.0625
Q3.2: HTHHH and TTHTT	0.15625	0. 15,625	0.3125	**0.4375**	0.375	0.375
Q3.3: HHHTT and HTTTH	0.3125	0.3125	0.3125	0.3125	0.25	**0.375**

In the first question (Q3.1), HTHTH and HHHHH have the same probability of occurring when the sample space is partitioned by longest run or alternations (see Table 3). These sequences, however, do have a different probability of occurring when the sample space is partitioned by the ratio of heads to tails. That is, HTHTH is more likely than HHHHH as having 2 tails (HTHTH) is more likely than no tails.

Similarly, in the second question (Q3.2), HTHHH and TTHTT are equally likely to occur under the sample space of the ratio of heads and tails and the sample space of the number of alternations. However, a longest run of three (HTHHH) is less likely than a longest run of two. In the third question (Q3.3), HHHTT and HTTTH are equally likely to occur under the sample space of the ratio of heads and tails and the sample space of the longest run. However, one alternation (HHHTT) is less likely than two alternations.

This means, along with selecting the response that the sequences are equally likely, a participant who suggests *HHHHH is least likely to occur because there are no T’s, HTHHH is least likely to occur because it has three heads in a row, and HHHTT is least likely to occur because it swaps from heads to tails too infrequently* would also be correct if these alternative sample spaces had been used. While 13 participants responded non-normatively to Q3.1, and four participants responded non-normatively to Q3.2 and Q3.3, only one participant answered all three questions according to the correct alternative sample space responses (Tables 4, 5, and 6). It may be that one participant potentially implemented alternative sample spaces, but further investigation into their underlying thinking would be needed in order to confirm this. Furthermore, supporting the case that alternative sample spaces may have been used, this participant answered all other questions relating to the representativeness heuristic normatively. Internal validation may therefore suggest that their responses in Q3.1–Q3.3 are not indicative of heuristic thinking, although, as noted, more in-depth exploration into the participant’s thinking would be necessary to validate this claim.

**Table 4:** Frequency of responses to question 3.1

Which is least likely?	Frequency
HTHTH alternates too frequently	0
HHHHH has no alternations	5
HTHTH is too patterned	1
HHHHH has no T’s	7
They are both as likely to occur	137
I’m not sure	0

**Table 5:** Frequency of responses to question 3.2

Which is least likely?	Frequency
HTHHH has three heads in a row	1
TTHTT is too patterned	3
They are both as likely to occur	146
I’m not sure	0

**Table 6:** Frequency of responses to question 3.3

Which is least likely?	Frequency
HHHTT alternates too infrequently	1
HTTTH is too patterned	2
They are both as likely to occur	146
I’m not sure	1

**Fig. 2:**
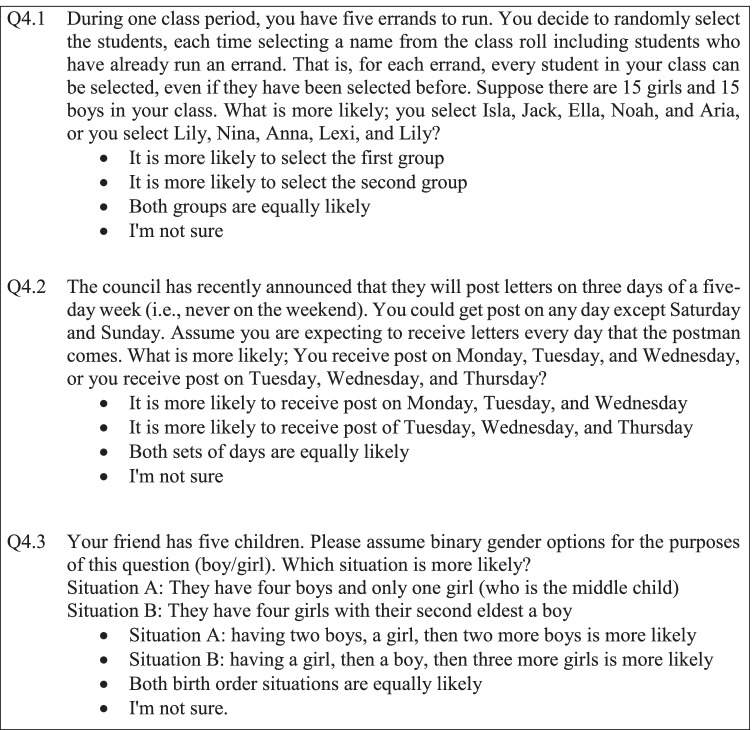
Three context-equivalent comparison questions from the questionnaire

Understanding participants’ underlying thinking from their responses to these questions is still limited. In particular, those who suggested the sequences in each question were equally likely may recognise this is the “correct” answer but still have randomness misconceptions. Coin-toss sequences are quite a familiar context, commonly explored in research on the representativeness heuristic. To assist with internal validity, the participants were also asked context-equivalent questions. There were some difficulties with the context-equivalent questions, with the potential of some participants being distracted by the particulars of the context (particularly with the post example, Fig. 2). To ensure comparability to the coin-toss questions, these were not necessarily practical real-world contexts. Q4.1 (GBGBG vs. GGGGG, G—girl and B—boy) is comparative to Q3.1 (HTHTH vs. HHHHH); Q4.2 (PPPNN vs. NPPPN, P—post received in the mail and N—no post received in the mail) is comparative to Q3.3; and Q4.3 (BBGBB vs. GBGGG, G—girl and B—boy) is comparative to Q3.2. The probabilities for each set of sequences for the alternative sample spaces can be seen in Table 7.

**Table 7:** The likelihood of sequences 1 and 2 for questions 4.1, 4.2, and 4.3 under three alternative sample spaces, with the more likely sequence identified in bold

	Sample space partition					
	Ratio of heads and tails		Longest run		Number of switches	
	Sequence 1	Sequence 2	Sequence 1	Sequence 2	Sequence 1	Sequence 2
Q4.1: GBGBG and GGGGG	**0.3125**	0.03125	0.0625	0.0625	0.0625	0.0625
Q4.2: PPPNN and NPPPN	0.3125	0.3125	0.3125	0.3125	0.25	**0.375**
Q4.3: GBGGG and BBGBB	0.15625	0. 15,625	0.3125	**0.4375**	0.375	0.375

The four participants who selected non-normative responses in Q3.1–Q3.3 suggested the scenarios were equally likely in all of Q4.1–Q4.3. Only one participant selected a non-normative response for a question in coin-toss comparison and context-equivalent questions (Q3.1 and Q4.2, which are not an equivalent pair).

As in Table 8, seven participants answered Q4.1 non-normatively, all suggesting the first group was more likely (GBGBG). However, as the second sequence has “Lily” twice and even though the question specifies that this random selection is with replacement, participants could have misread the question, misunderstood the effect of selection with replacement, had issues with independence, and/or rejected non-equal ratio of heads to tails.

**Table 8:** Frequency of responses to question 4.1

Which is most likely?	Frequency
The first group (GBGBG)	0
The second group (GGGGG)	7
Both groups are equally likely	142
I’m not sure	0

There is also potentially confusion around Q4.2 as the context strayed from a practical, real-world scenario. The idea that the probability you receive post through the mail is equal to the probability you do not receive post through the mail diverges considerably from real-world expectations. Nine participants were unsure while five participants selected each of the two non-normative options as seen in Table 9. By comparison, Q4.3 is posited as being a more familiar context as it relates to birth order. As in Table 10, only five participants answered non-normatively to Q4.3. Four participants felt situation A was more likely (BBGBB) and only one participant felt situation B was more likely (GBGGG).

**Table 9:** Frequency of responses to question 4.2

Which is most likely?	Frequency
Monday, Tuesday, and Wednesday (PPPNN)	5
Tuesday, Wednesday, and Thursday (NPPPN)	5
Both sets are equally likely	129
I’m not sure	9

**Table 10:** Frequency of responses to question 4.3

Which is most likely?	Frequency
Situation A (BBGBB)	4
Situation B (GBGGG)	1
Both sets are equally likely	145
I’m not sure	0

The difference between responses to Q3.1–3.3 and Q4.1–4.3 highlights the value in attempting to reveal more about participants’ underlying thinking. While an attempt was made to bridge between quantitative and qualitative responses through the use of descriptive multiple-choice options, it remains impossible to tell whether participant responses were due to heuristic thinking or valid reasoning. For example, participants who selected non-normative responses may have been attempting to use alternative sample spaces but failed to correctly identify which sequence was more likely as the calculations were not provided. Likewise, there may be participants who selected the equally likely option because familiarity means they recall the correct answer rather than this response originating from a correct understanding. Valuable insight into participants’ interpretation of the question and underlying thinking is hence lost through the lack of data from verbal communication and researcher probing.

As per Shaughnessy (1977), it is necessary to query participant thinking to obtain more accurate data. This is most easily done through interactive discussions. However, the impact of Covid-19 on the availability of potential participants for the thesis study meant that interviews could not be held in-person or with enough flexibility to fit to teachers’ very busy pandemic lives. Adapting to the pandemic meant our population of interest were overwhelmed with continuing to provide education while navigating unfamiliar environments. As a result, to maximise data collection, we used a survey method rather than online interviews so the participants could complete the questionnaire at a time that best suited them.

## Further Examples

To further exemplify that alternative sample spaces may provide an appropriate reason for responses of not-equally-likely to coin-toss (and similar) sequence questions, we now explore various sequence comparisons found in two studies from the literature. This follows the pilot study and thesis study as supplementary commentary that further demonstrates how alternative sample spaces could feasibly be used by participants in additional contexts.

### Pfannkuch and Brown (1996)

In their empirical investigation, Pfannkuch and Brown (1996) interviewed five female psychology students. One question referenced a sequence from a roulette wheel. The question asked: *A gambler observes the ball to land on red six times in a row, that is RRRRRR. What do you expect the next colour to be?* (p. 3). There are two possible outcomes from the next spin of the wheel, either red or black, so we are comparing the following two sequences:

Sequence 1: RRRRRRRSequence 2: RRRRRRB.

This is quite a short sequence, with only seven observations, so it is possible that participants could have judged these according to their long run expectations (i.e., a more equal number of R and B). There are ${2}^{7}=128$ possible sequences. Similar code to that shown in the Appendix was used to calculate the following:

- Considering a sample space of the number of heads/tails, there is only one sequence of seven R $\left(\frac{1}{128}=0.00781\right)$ but seven sequences with six R $\left(\frac{7}{128}=0.0547\right)$. So, sequence 2 is more likely.
- Considering a sample space of the length of the longest run, there are two sequences with a longest run of seven $\left(\frac{2}{128}=0.0156\right)$ but four sequences with a longest run of six $\left(\frac{4}{128}=0.0313\right)$. So, sequence 2 is again more likely.
- Considering a sample space of the number of switches, there are two sequences with zero switches $\left(\frac{2}{128}=0.0156\right)$ but twelve sequences with one switch $\left(\frac{12}{128}=0.0938\right)$. So, sequence 2 is again more likely.

While it is feasible that participants who selected sequence 2 as being more likely were using an alternative sample space, it is only after reflecting on the verbal explanations that researchers can uncover whether this was actually the case. In the example provided in the paper, one participant initially suggested that the next colour would be black, before changing this to red or black being equally likely. However, when probed about their initial response, the participant reasoned the next colour would be black: “Because there’s all red that have come out so it must be time for a black to come out” (p. 4). From this reasoning, we can suggest that the participant was unlikely to be using an alternative sample space. Their reference to “…it must be time…” may imply that they are expecting a balance of red and black. This may indicate use of the representativeness heuristic (Kahneman & Tversky, 1972, 1974). However, from the transcript provided, it is difficult to determine whether this is actually the case. While indicative of misconceptions, deeper exploration into the participant’s reasoning may provide more insight. Could it be possible that the participant judged the sequences using one of these alternative sample spaces but failed to express this? Could the participant’s intuition be based on an alternative sample space without their awareness? These questions would require further research to be answered.

### Batanero et al. (1996)

In a study investigating the perceptions of high school students, one question asked the following:

Item 1. Which of the following sequences is more likely to result from flipping a fair coin 5 times:

(a) HHHTT; (b) HTTHT; (c) THTTT; (d) HTHTH?

Similar code to that shown in the Appendix was used to calculate the following:

- Considering a sample space of the number of heads/tails, the probability of three heads is $0.313$, the probability of two heads is also $0.313$, and the probability of one head is $0.156$. So, all sequences are equally likely, with the exception of option (c).
- Considering a sample space of the length of the longest run, the probability of a longest run of three is $0.313$, a longest run of two is $0.4375$, and the probability of a longest run of one is $0.0625$. So, option (b) is the most likely.
- Considering a sample space of the number of switches, the probability of one switch is $0.25$, two switches is $0.375$, three switches is also $0.25$, and the probability of four switches is $0.0625$. So, option (b) is again most likely.

The authors commented that “… sequence b may appear more representative than the others” (p. 53), and they conjecture that the selection of option (b) may be indicative of this. However, if alternative sample spaces were used, it is not unreasonable for participants to have selected option (b) as more likely if the length of the longest run or number of switches had been considered. The participants in this study responded to a questionnaire and were not interviewed, meaning that the use of alternative sample spaces can only be inferred, not substantiated.

## Conclusion

From the research by Chernoff (2009) and Shaughnessy (1977, 1992), attempting to reveal participant thinking and reasoning may provide insight into the use of alternative sample spaces. The two participants in the pilot study may have held a non-normative perspective and their selection of sequence may have been correct under an alternative sample space. However, there is insufficient evidence to conclude that this participant was using alternative sample spaces and the others were not.

As noted, the adoption of alternative sample spaces can lead to one sequence being more likely than another. Whether an alternative sample space was used by the participants in the pilot and thesis studies is unclear, and further exploration would have been necessary to obtain a clearer picture of the participants’ potential thought processes. Even with further probing, the participants’ true reasoning behind their choices may have still be uncertain—*were the participants aware of their own thinking? Would they have been able to further articulate their thinking?*

In considering the pilot study participants’ verbal reasoning, we can only infer possible meaning based on what is articulated; our analysis of which, as put by Leron and Hazzan (1997), may “…fall short of describing the student’s mind in all its richness and complexity” (p. 266). As much as we may like to have a transcript of students’ mental processes, we are limited to what we see, what we hear, and how we interpret this.

While the use of alternative sample spaces was the focus of this paper, there are numerous other aspects that could have influenced the participants’ choices, such as the lack of an “equally likely” option in the pilot study, the participants’ understanding of the questions in both the pilot and thesis studies, and the degree to which participants contemplated the possible answers (i.e., they might have simply guessed!).

Research evaluating participant responses has developed since Tversky and Kahneman (1974), and while research such as this paper further highlights components to consider when undertaking this activity, there are still many aspects requiring refinement to improve inference robustness. Revealing participants’ underlying thinking is a challenging endeavour requiring careful implementation of tasks, and although true thought processes may remain ambiguous, continued improvement in research execution can help researchers to establish more comprehensive conclusions.
